# Long-Term Follow-Up After Bariatric Surgery: Key to Successful Outcomes in Obesity Management

**DOI:** 10.3390/nu16244399

**Published:** 2024-12-21

**Authors:** Aleksandra Budny, Agata Janczy, Michal Szymanski, Adriana Mika

**Affiliations:** 1 Department of Pharmaceutical Biochemistry, Faculty of Pharmacy, Medical University of Gdansk, 80-211 Gdansk, Poland; a.budny@gumed.edu.pl; 2 Division of Food Commodity Science, Faculty of Health Sciences with the Institute of Maritime and Tropical Medicine, Medical University of Gdansk, 80-211 Gdansk, Poland; agata.jonczy@gumed.edu.pl; 3 Division of Oncological, Transplant and General Surgery, Faculty of Medicine, Medical University of Gdansk, 80-214 Gdansk, Poland; szymanski@gumed.edu.pl; 4 Department of Environmental Analytics, Faculty of Chemistry, University of Gdansk, 80-308 Gdansk, Poland

**Keywords:** obesity, bariatric surgery, obesity management, follow-up, eating disorders, nutrition management

## Abstract

**Background/Objectives**: Bariatric surgery (BS) is considered one of the most effective interventions for the treatment of obesity. To achieve optimal long-term results, continuous follow-up (FU) within a multidisciplinary treatment team is essential to ensure patient compliance and maximize the benefits of BS. However, many patients find it difficult to maintain regular FU, which can affect the quality of care and lead to postoperative complications. This review aims to highlight factors that may hinder compliance with FU after BS, examine potential causes and consequences of inadequate FU, and identify strategies to improve patient participation in long-term FU. **Methods:** The literature search was conducted between October 2023 and June 2024 in Medline (PubMed) and the Cochrane Library datasets. Studies were selected for their relevance to adherence to FU, multidisciplinary approaches, and long-term bariatric outcomes. **Results:** The pre- and postoperative period is critical for educating patients and healthcare team members about the importance of FU, addressing potential barriers (e.g., logistical, psychological, and social challenges), and highlighting the risk of relapse to obesity after surgery. The lack of a standardized FU protocol leads to differences between medical centers, further impacting patient adherence. **Conclusions:** Tailored and regularly updated strategies are essential to address individual patient needs and improve adherence to FU. Further research is needed to identify the specific factors that influence variability in long-term BS outcomes, highlighting the need for a patient-centered approach to obesity treatment.

## 1. Introduction

The World Health Organization (WHO) reports that the prevalence of obesity is currently increasing at an alarming rate worldwide. Currently, over 1.9 billion people in the world are overweight, and 650 million are obese [1]. In addition to a higher mortality risk, obesity is associated with numerous comorbidities such as dyslipidemia, insulin resistance, type 2 diabetes (T2D), circulatory disease, hypertension, and obstructive sleep apnea [2].

Bariatric surgery (BS) is currently the only method with proven long-term effects as measured by weight loss (WL) and remission of comorbidities, which is why it forms the basis of obesity therapy [3]. However, it should be accompanied by lifestyle changes, including an appropriate diet and regular physical activity (PA). The multidisciplinary team preparing the patient should include, for example, a psychologist, a dietitian, and a physiotherapist [4]. Bariatric patients are encouraged to change their eating habits to better adapt to the dietary changes required after surgery. WL reduces the risk of perioperative complications [5]. Surprisingly, malnutrition is common in patients with obesity, so the preoperative diet should be prepared in collaboration with a dietitian [6]. Early revision, which is recommended in the Enhanced Recovery After Bariatric Surgery (ERABS) protocols, aims to reduce the risk of perioperative complications and shorten the length of hospital stay [1]. After surgery, the patient goes through several dietary phases, and in order to achieve the planned results, the dietary recommendations must be followed consistently and permanently. This is important because BS aims to reduce weight and maintain WL by restricting food intake (limiting gastric capacity), malabsorption of food (limiting nutrient intake), or through a neurohormonal response that regulates hunger and energy balance. Some surgical interventions are a combination of these two methods (restrictive and malabsorptive) [7,8,9]. In addition, BS leads to changes in digestion and absorption in the gastrointestinal tract, which can result in protein, micronutrients, and vitamin deficiencies. To avoid the consequences of deficiency symptoms, adequate nutritional supplementation is extremely important.

Dietary recommendations and changes in digestion and absorption in the gastrointestinal tract after BS can influence the level of laboratory parameters reflecting metabolic and nutritional status [10,11]. Therefore, the path to a successful outcome goes beyond the surgical procedure itself and requires a comprehensive approach to lifestyle management. This review addresses the critical aspects of postoperative lifestyle modifications that are essential for optimizing patient outcomes. Particular attention has been paid in this review to the role of nutrition, PA, psychological support, pharmacologic treatment, and long-term follow-up in improving the effectiveness of BS. The aim of this review is to provide a comprehensive framework for healthcare providers to support long-term success throughout the patient’s treatment process.

## 2. Obesity as a Widespread Disease

**Figure 1:**
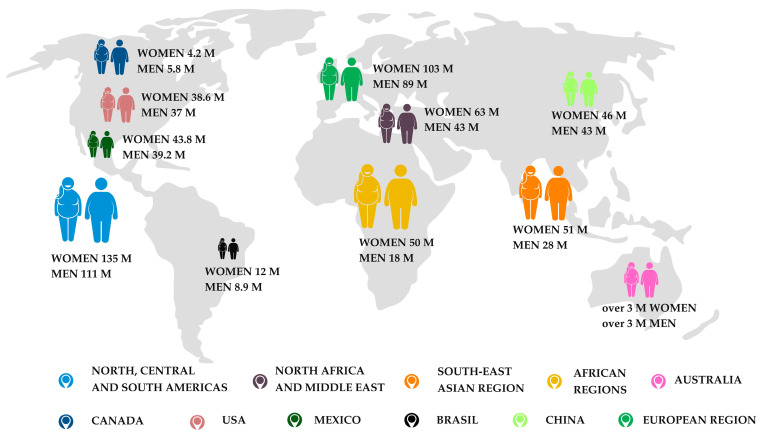
Comparison of obesity prevalence between world regions from 2020 based on [19,20,21,22,23].

The number of people with obesity in the world is expected to rise to over 1 trillion by 2025. According to data from 2019, 103 million women and 89 million men in Europe suffer from obesity (Figure 1). Sedentary behavior, i.e., a decrease in outdoor activities and an increase in screen time, had a negative impact on mental and physical health, including weight gain and a deterioration in nutritional status [12]. In order to diagnose obesity, the BMI is calculated. It is an indirect marker of obesity and allows obesity to be categorized into three levels of severity, including Class I—BMI ≥ 30 kg/m^2^, Class II—BMI ≥ 35 kg/m^2^, and Class III—BMI ≥ 40 kg/m^2^ [13]. The World Obesity Federation predicts that by 2030, almost one in three men (29.42%) and one in three women (29.97%) in Europe will have a BMI of ≥30 kg/m^2^. This equates to approximately 102 million men and 113 million women in the region at risk of obesity-related complications by 2030 [13]. It is worth noting that the COVID-19 lockdown has led to significant environmental and social changes in people’s lives, which in turn have impacted weight changes [12]. According to the Ipsos COVID 365+ study, 42% of people in Poland have gained an average of 5.7 kg [14] [11]. In addition, obesity and metabolic diseases are considered determinants of severe COVID-19 and mortality [15]. About 20% of all patients hospitalized for COVID-19 suffered from obesity alone [16]. The combination of COVID-19 and the obesity pandemic has highlighted the importance of obesity prevention and control and better systemic solutions.

Obesity not only affects physical and mental health but also has numerous economic consequences for national healthcare systems [17]. Currently available treatments for obesity include behavioral changes (e.g., diet, physical activity) as well as psychological, pharmacologic, and surgical interventions [18].

## 3. Bariatric Surgery

In people struggling with severe obesity (SO), lifestyle changes and pharmacotherapy alone may not be sufficient to achieve significant and lasting WL [24,25,26,27]. In such cases, BS offers a promising solution [28]. According to the International Federation for the Surgery of Obesity and Metabolic Disorders (IFSO), the various bariatric procedures include a range of surgical interventions designed to help reduce weight and promote long-term health. Each procedure has its advantages, disadvantages, and long-term results. BS is a group of surgical procedures aimed at achieving significant weight loss in people with severe obesity. By remodeling the digestive system, BS not only helps reduce calorie consumption but also treats the comorbidities associated with obesity.

The changes brought about by BS have a significant effect on the digestion and absorption processes. A smaller stomach limits the amount of food that can be absorbed, promoting an early feeling of fullness. In addition, by rerouting or shortening the intestine in certain procedures, the surface area for nutrient absorption is reduced, changing the way the body processes and absorbs nutrients.

These structural changes are accompanied by hormonal shifts, particularly in gut hormones such as ghrelin and glucagon-like peptide-1 (GLP-1) [29,30], which regulate hunger, satiety, and metabolism. The reduction in gastric acid production and digestive enzyme activity combined with changes in the gut microbiota further influence digestion and nutrient absorption.

The most frequently performed bariatric procedures include:

Sleeve Gastrectomy

Sleeve Gastrectomy (SG) was first described in 1993 as a variant of biliopancreatic diversion [31]. In this procedure, a large portion of the stomach is removed, leaving a smaller “sleeve” shape [32]. This stand-alone bariatric procedure is now the most commonly performed BS procedure in the world [33]. The amount of food that can be eaten is limited, and the WL is thus supported. SG is not only a restrictive procedure but also has metabolic effects. In addition to reducing the size of the stomach, hormones, and appetite are also regulated, which can contribute to a further improvement in WL and metabolism [34,35]. After SG, ghrelin levels drop significantly, which leads to a reduced appetite and a faster feeling of fullness after meals. This hormonal change contributes to long-term WL [36]. In addition, SG also influences the production and secretion of other hormones, such as peptides YY and GLP-1, which are known to promote satiety and regulate blood glucose levels. These hormonal changes help to curb hunger, control blood sugar, and improve overall metabolic function [30]. A long-term observational study by Diamantis et al. reported that the average percentage excess weight loss (%EWL) after SG was over 50 for 5 or more years [37]. In the recent publication by Çeler et al. [38], the remission of T2D in the postoperative month was 71.1%. Weight regain (WR) and the development of recurrent gastro-oesophageal reflux disease (GERD) remains a major problem after SG and may require reoperation [39,40]. The causes of WR can be multifactorial and include factors such as changes in dietary habits, decreased physical activity, and hormonal changes [41,42].

One Anastomosis Gastric Bypass (OAGB)

The One Anastomosis Gastric Bypass (OAGB), also known as the Mini-Gastric Bypass (MGB), is another bariatric procedure that has gained popularity in recent years [40]. The OAGB/MGB was first introduced by Robert Rutledge in 1997. In this operation, the stomach is reshaped to create a small and long pouch, similar to an SG, but with a single anastomosis to the small intestine approximately 180 cm distal to the Treitz ligament. Reducing the size of the stomach restricts food intake, and rerouting the digestive system leads to changes in gut hormones, resulting in improved metabolism and reduced caloric intake [43]. The procedure has shown promising results in terms of significant post-operative weight loss (post-op WL) and improvement in obesity-related conditions, such as T2D, hypertension, and obstructive sleep apnoea syndrome [44,45]. However, like any bariatric procedure, OAGB/MGB is not without potential risks and complications [46]. Potential complications include dumping syndrome, bile reflux, ulcers, nutritional deficiencies (ND), and long-term complications related to altered anatomy associated with SIBO (small intestinal bacterial overgrowth) [47,48,49].

Roux-en-Y Gastric Bypass

RYGB was first introduced by Edward E. Mason in 1966 and has since been established, with some modifications, as an effective treatment for obesity and related health disorders [50,51]. In RYGB, a small gastric pouch is created by dividing the upper part of the stomach. The small pouch is then connected directly to the small intestine, bypassing part of the stomach and duodenum. This altered anatomy restricts the amount of food that can be ingested and limits calorie intake [52,53]. Over the years, RYGB treatment has established itself as the gold standard among bariatric procedures, as it has been shown to provide significant and sustained WL [28]. Long-term studies have shown that RYGB can lead to an average WL excess of 60–80% in 5 years after the procedure [54,55]. Like any surgical procedure, RYGB has potential risks and complications. These can include postoperative complications such as leakage, bowel obstruction, and ND in the long term [56,57,58].

Single Anastomosis Sleeve Ileal Bypass with Sleeve Gastrectomy (SASI-S)

Single Anastomosis Sleeve Ileal Bypass with Sleeve Gastrectomy (SASI-S) is a procedure that combines aspects of sleeve gastrectomy and intestinal bypass to achieve WL and metabolic improvement. It is a modification of the Santoro operation [59], which was introduced in 2016 by Tarek Mahdy [60]. SASI-S combines the benefits of restricted food intake with preserved movement of food through the entire gastrointestinal tract. It utilizes the efficient passage of undigested food through the ileum, triggering the release of satiety hormones. One year after surgery, higher post-op WL, lower BMI, and higher %EWL were observed compared to the SG cohort [61] and the OAGB cohort [62]. Currently, there is limited information on the long-term effects of SASI-S, specifically on biliary reflux [63].

## 4. Preparation for Surgery

For a successful BS, it is important to assess the patient’s psychological, nutritional, and functional health status during the preoperative assessment [64]. This process requires the collaboration of various specialists, including bariatric surgeons, dietitians, psychologists, physiotherapists, bariatric nurses, and the patients themselves, in accordance with the ERABS guidelines [1,65,66]. These guidelines recommend evidence-based strategies to reduce perioperative stress and support postoperative physiologic function. The use of multiple interventions during patient care has been shown to improve outcomes, including reducing complication rates and shortening hospital length of stay. The elements of modern care can be divided into three distinct categories: preoperative interventions aimed at changing lifestyle and optimally preparing the patient for surgery, perioperative interventions aimed at reducing the stress caused by surgery, and postoperative interventions aimed at accelerating convalescence. The latest recommendations on prehabilitation were presented in Poland in 2023. These guidelines, which focus on the comprehensive preparation of patients for surgery, set out the basic principles of pre-habilitation and the methods for its implementation. The document aims to standardize these recommendations. In the context of BS, prehabilitation includes four important components: nutrition, physical therapy, psychological preparation, and the removal of dependencies [66].

Nutritional intervention should be individualized for each patient, with the goal of improving health outcomes by reducing the risk of chronic disease and improving quality of life, rather than focusing solely on WL. Tailoring the nutritional plan to the patient’s clinical condition and preferences increases the likelihood of long-term adherence to treatment [67,68]. The basis of nutritional treatment for obesity is to reduce caloric intake, but diets that excessively restrict caloric intake or are unbalanced are not recommended. Obesity treatment should focus on food quality and developing a healthy relationship with food. This includes practices such as mindful eating, which can help reduce cravings, emotional eating, and overeating while improving body satisfaction and awareness of hunger and satiety [68]. Certain groups of patients undergoing BS may require special attention to food preparation. These include patients with extremely high BMI, sarcopenic obesity, ND, poor glycemic control, untreated eating disorders (ED), or limited knowledge of nutrition. While various methods for managing these patients have been described in the literature, in practice, a more detailed assessment of nutrition and nutritional status is often required, along with personalized interventions prior to surgery [69].

In the preoperative phase, approaches such as a low-calorie diet (LCD), a very low-calorie diet (VLCD), or immunonutrition (IMN) may be considered. This choice often depends on the surgical center. LCD and VLCD are the most commonly used short-term pre-BS diet plans. These diets are designed to partially or completely replace regular meals and usually supplement one or two standard meals per day. The LCD diet usually provides 800–1200 kcal per day, while the VLCD diet ranges between 500 and 800 kcal per day [70]. These diets are used in patients with a BMI greater than 35 kg/m^2^ when a rapid WL is required prior to surgery or when a standard low-calorie diet (1200–1500 kcal) has not provided adequate results [71]. 

An IMN, in which certain nutrients are administered in quantities that exceed basic requirements in order to strengthen the immune system, can also be beneficial. Nutrients such as arginine, omega-3 fatty acids, and glutamine can reduce the risk of perioperative complications and accelerate wound healing and recovery 72 IMN has a positive effect on the treatment of underlying diseases and comorbidities and, at the same time, helps to stabilize the patient’s nutritional status [66]. 

To simplify the patient’s daily life, meals should be easy to understand and follow. The Bariatric Plate Model is an effective tool to teach patients how to consume macronutrients and micronutrients in a balanced way to lose weight and maintain a healthy diet in the long term [72]. Half of the plate (50%) should consist of protein-rich foods, such as beef, chicken, fish, eggs, low-fat dairy products, and legumes. About a third (30%) should consist of vitamins, minerals, and fiber from fruits to vegetables, while the remaining portion should include carbohydrates such as whole grains, pasta, rice, and potatoes. Patients should also remember to include healthy fats such as olive oil, rapeseed oil, nuts, seeds, or kernels in every meal. In addition, it is important to ensure adequate fluid intake, take daily vitamins, minerals, and proteins, and exercise regularly [73].

**Figure 2:**
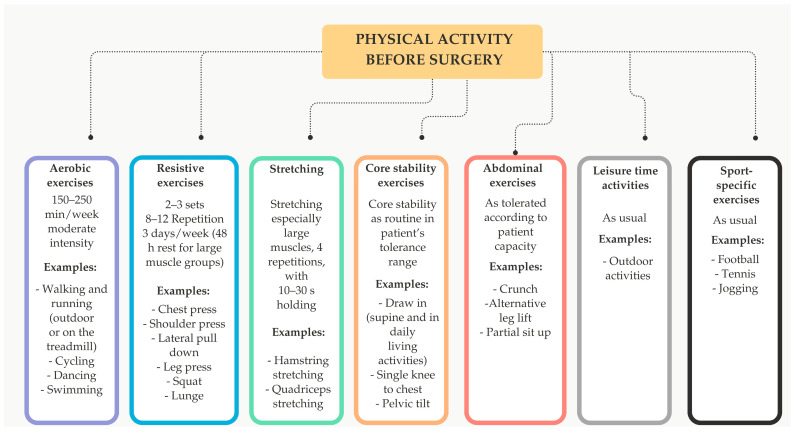
Physical activity before surgery based on [69].

Every adult who is overweight or obese should consider increasing physical activity, as it is an essential part of both obesity treatment and a healthy lifestyle [68]. Regular PA helps to reduce intra-abdominal fat, increase lean mass (muscle and bone), and counteract the decline in resting energy expenditure caused by WL. It also lowers blood pressure, improves glucose tolerance, insulin sensitivity, and lipid profile, increases physical fitness, and supports long-term weight maintenance. In addition, it also increases well-being and self-esteem and reduces anxiety and depression [8]. This is particularly important, as many people in this group have little experience designing their own exercise routines. The goal of exercise programs is to promote a habit of daily PA that lasts a lifetime. Consistent PA not only contributes to a sustainable WL but also helps to maintain the target weight in the long term. Currently, there are no standardized guidelines to support BS in developing a balanced exercise program that addresses cardio-respiratory fitness, strength, and flexibility. This is particularly important as this patient group often lacks experience in designing their own exercise program. It is recommended to engage in at least 150 min of moderate to vigorous exercise per week—equivalent to 3–6 METs (metabolic equivalents) or approximately 10,000 steps per day—to improve overall health. This should include strength training 2–3 times per week that targets all major muscle groups to maintain lean body mass, improve flexibility, and prevent muscle loss during WL [3,8,68,74]. Optimal results are achieved when exercise recommendations are precise, tailored to the individual’s health and physical abilities, and consistent with current guidelines. Several factors should be considered: (1) the type of exercise (e.g., endurance, strength, or mixed), (2) the frequency (number of sessions per week), (3) the dose (duration of activity in minutes), (4) the fitness (e.g., cycling, swimming, or Nordic walking), and (5) the intensity (e.g., heart rate during exercise) [68]. The type and intensity of PA for bariatric patients correspond to the recommendations for the treatment of obesity (Figure 2) [69].

The psychological assessment for BS in people with SO aims to diagnose any comorbidities and initiate the necessary treatment to optimally prepare the patient for surgery and improve outcomes. The primary goal of this assessment is to determine if there are psychological contraindications to BS. It is important to note that psychological disorders do not automatically preclude a patient from surgery; rather, the patient’s mental status should be stabilized prior to surgery [75]. In addition, psychological support should be offered both before and after surgery to improve long-term success and reduce the risk of complications. All factors contributing to the persistence of obesity need to be identified and addressed prior to surgery [76]. The main goals of the preoperative psychological assessment are to identify contraindications and obstacles in the preoperative phase, to recognize factors that may predict early weight regain, to select patients who need special psychological support before and after surgery, and to identify potential postoperative challenges. In addition, this assessment helps develop strategies to support behavioral changes that can improve the safety and effectiveness of surgery as well as long-term weight management. If a patient does not meet the criteria for surgery, alternative treatment options can be recommended [77]. The most commonly used psychological intervention is cognitive behavioral therapy (CBT). There is also promising evidence for the effectiveness of interventions based on Acceptance and Commitment Therapy (ACT) and Dialectical Behavior Therapy (DBT) [69,77].

## 5. Obesity Therapy in the Context of Surgical Treatment

Inadequate post-op WL is attributed to a return to inappropriate lifestyle habits, noncompliance with the postoperative diet, or lack of patient knowledge, and not necessarily to surgical or metabolic causes [42]. The use of multiple interventions during patient care has been shown to improve outcomes, including reducing complication rates and shortening hospital length of stay. The elements of modern care can be divided into three distinct categories: preoperative interventions aimed at modifying lifestyle and optimally preparing the patient for surgery, perioperative interventions aimed at reducing the stress caused by surgery, and postoperative interventions aimed at accelerating convalescence. A patient who is a candidate for BS should be well informed about the changes that occur after BS and their consequences [1]. They must be motivated and willing to participate in long-term care, change their eating habits, and adopt a modified lifestyle after surgery. To ensure realistic expectations, a preoperative education program is often recommended to reduce anxiety, wound complications, postoperative pain, and length of hospital stay. Apart from the surgical benefits, this is an informative factor for the surgeon and the bariatric team, demonstrating the patient’s determination to change their lifestyle. For this purpose, an LCD or VLCD is recommended for 2–4 weeks. It is emphasized that the data on the influence of pre-operative weight loss (pre-op WL) on the final bariatric effect are contradictory in the available literature. However, there is no doubt that a pre-op WL of 10% is associated with a lower risk of perioperative complications and shortens the length of hospital stay. In the perioperative period, it is recommended to consume solid food (equivalent to a light meal) for at least 6 h and clear liquids for 2 h before anesthesia is induced, unless there are contraindications. However, if nutrition is secured early, the patient should consume clear oral fluids and ONS (oral nutritional supplements) on the first day after surgery [1].

On the other hand, studies show that mental health deteriorates in some people after BS. Komorniak et al. investigated the effects of probiotics, microbiota composition, and dietary changes on the mental health of patients who had mood disorders after BS. Probiotics and an improvement in dietary habits can improve the mental health of people suffering from mood disorders after BS [78]. In the study by Depommier et al., a 3-month supplementation with *Akkermansia muciniphila* significantly improved the metabolic parameters of patients with obesity [79]. Probiotics can therefore not only support the composition of the gut microbiota of patients before and after BS but also change the level of inflammation and the mental state of patients. However, it should be kept in mind that targeted probiotic therapy is required, adapted not only to the patient’s health status but also to their diet or type of surgery.

### 5.1. Nutrition After Bariatric Surgery

The following approaches to nutritional management of the postoperative bariatric patient are applicable to most patients. Nutritional counseling should include advice on texture progression depending on the surgical procedure (e.g., size of residual stomach, presence of gastrojejunostomy, etc.) and the practices of the bariatric center [1]. Nutrition following a BS serves a dual purpose. First, adequate energy and nutrients are needed to support post-BS tissue healing and promote the maintenance of lean body mass during extreme WL. Second, the foods and beverages consumed post-BS must minimize reflux, dumping syndrome, and early satiety while maximizing WL and ultimately weight maintenance [80]. In general, the postoperative diet after BS is divided into several phases, depending on the consistency and amount of food the patient can tolerate (Table 1) [3,81,82]. According to the ERABS protocol, a clear liquid meal can be started a few hours after surgery before moving on to nutritious liquids [1]. Kushner et al. recommend starting solid, soft foods 10–14 days after BS once the gastrointestinal tract has healed, as do the 2019 AACE/TOS/ASMBS/OMA/ASA guidelines [3]. Patients should eat a liquid, protein-rich diet, e.g., protein-rich natural yogurt, creamy soups, and protein-rich milkshakes that are not very sweet. Soft, solid foods are included in the diet, with an emphasis on protein sources, some carbohydrates, and fiber (e.g., fruits and vegetables). At this stage, it is not necessary for most patients to puree their food unless they still have difficulty chewing. However, there is no gold standard for food expansion. Each bariatric center has its own nutritional guidelines for patients. Usually, a later introduction of solid meals is recommended about 4 weeks after BS [3]. In a Dutch study, Theunissen et al. introduced a new dietary protocol in 936 patients undergoing RYGB surgery and allowed the transition to solid food 12 h after surgery. All patients received detailed dietary instructions and advice on eating behavior from a qualified dietitian and psychologist prior to surgery. At 30 days post-BS, the complication rate was 9.4% and only 0.6% had a gastrointestinal. They concluded that an early transition to solid food is a viable alternative, as no increased complication rate was observed [83]. This may confirm that it is possible to accelerate the transition to solid meals if the patient is well guided by specialists. It should also be emphasized that it is not advisable to eat meals with altered consistency for too long, as comminuted food leaves the stomach faster and does not produce a long-lasting feeling of satiety, so the feeling of wanting to eat the next portion occurs faster after eating these foods than after solid food. After 4 weeks, patients can start eating meals without changing the consistency of the products. Of course, the intake can be shortened as well as extended due to individual tolerances and conditions. Patients who have problems prolonging their diet due to fear of pain, vomiting, and nausea should seek advice from a qualified dietitian. During this phase, it is very important to advise patients to eat slowly, chew their food thoroughly, stop eating as soon as they are full, and not to eat and drink at the same time. All patients should be educated about their postoperative nutritional needs. This includes guidelines for daily intake of adequate amounts of clear fluids to maintain water balance and urine output. Surgical patients should be provided with educational materials, shopping lists, and sample meal plans to help them adhere to the recommendations. In addition, patients should eat a balanced diet with a solid consistency for 6–8 weeks after surgery [81]. It is impossible to determine the exact size of a meal portion after BS. In this phase, it is usually recommended to eat meals with a volume of about 150 mL and gradually increase the portions to 200–250 mL (about 1 cup). The average food intake of operated patients is about 4–6 tablespoons per meal [72]. About 1 year after BS, patients are able to eat larger portions (200–250 g) [84]. A mandatory patient should stop eating when they feel full.

#### 5.1.1. Macronutrients

Dietary recommendations for macronutrients after LSG are similar to those after bypass surgery, although the exact requirements are not clearly defined and may need to be determined on an individual basis [69,81]. According to the latest guidelines, the typical daily calorie intake is 400 kcal/d in the first week after surgery and increases to 600 to 800 kcal/d in weeks 3 to 4 [3]. A systematic review of 8 studies found that energy intake increases over time beyond 1 year after surgery and ranges from 900 to 2425 kcal 2 years after BS [85]. The protein intake should then be individually adjusted to the patient’s gender, age, and body weight. The recommendations for protein intake after surgery range from 1.2 to 1.5 g/kg/day based on ideal body weight (at least 60 g protein/day for LSG/RYGB) [86]. A higher protein intake—up to 2.1 g/kg ideal weight per day—must be assessed on an individual basis [3]. In the first few weeks after BS, it is not possible to cover the majority of protein requirements with food. As protein deficiency and complications can occur in bariatric patients, they are often advised to take protein supplements to achieve the target daily intake [69]. In a randomized, controlled, double-blind pilot study, Schollenberger et al. indicate that protein supplementation after BS improves body composition by increasing the loss of body fat mass (FM) and decreasing the loss of lean body mass within the 6-month follow-up period [87]. It is recommended to consume at least 50 g per day at the beginning of the postoperative period and increase to 130 g per day as food intake increases. Patients should be encouraged to consume complex carbohydrates from sources such as whole fruits, vegetables, and whole grains. Simple sugars should be limited to less than 10 percent of daily calorie intake [81]. Data suggest that 40–45% of daily calories from carbohydrates from individual food sources and a fiber intake of about 14 g/1000 calories consumed would be appropriate according to BS [73]. In their review, Grosse et al. report a fiber intake of 10.4 to 11.7 g/day per person, which is well below the appropriate intake (25–30 g/day) [88]. The recommendations for fat intake after BS do not differ from those for the general population. Fat should make up about 20–35% of the daily calorie intake (35–60 g/d) [81,89,90].

One of the most difficult recommendations to follow after a BS is adequate water intake. This is mainly due to the limited gastric capacity, the difficulty in getting used to the principle of eating and drinking separately, but also to the changes in the sense of taste [81]. Avoiding sugary, caffeinated, and carbonated drinks as well as alcohol is indicated [82]. The recommended total fluid intake is 1500–1800 mL per day [69,81].

#### 5.1.2. Brief Recommendations for Nutrition

The current aim is to present the dietary recommendations to the patient as simply and clearly as possible. One method is to use the bariatric plate model. In addition, Table 2 summarizes the dietary recommendations according to BS. It also highlights the need to use the right quantity and quality of fats in the diet: olive oil, nuts, seeds, and kernels; drinking enough fluids; and taking supplements [73].

### 5.2. Long-Term Dietary Recommendations After Bariatric Surgery

According to the standards for bariatric care of the Section for Metabolic and Bariatric Surgery of the Association of Polish Surgeons, the postoperative phase begins on the 31st day after the procedure. Day after the surgery. During this period, patients should consume more and more solid products, and this phase of solid food should be maintained in the long term [91]. As the pace of dietary change after BS is individualized, some patients may require a diet with a different consistency for a longer period of time. However, as mentioned above, a dietitian should consider the specific needs of the patient when planning the diet. Around 6 months after surgery, the diet generally stabilizes, postoperative symptoms and food intolerances disappear, and patients eat larger meals [92]. After this phase, WL depends mainly on compliance with postoperative recommendations, and the effects of the mechanism of the surgery itself begin to diminish, especially in terms of reduction in gastric capacity but also, as some studies show, on hormonal changes (especially reduced secretion of ghrelin). In a study conducted 6 months after surgery, Sethi et al. found an increase in ghrelin levels compared to the study conducted 3 months earlier. Appetite, and thus the desire to eat certain foods, begins to play an important role in the postoperative period [93]. The reduced hunger lasted for over two years in some participants but only nine months in others. The desire to eat or try new foods also returned in many participants between six and 12 months after surgery [94]. It should therefore also be borne in mind that over time, appetite and thus the desire to eat certain foods begin to play a greater role than hunger itself. In view of this, most authors emphasize the need for long-term dietary monitoring [95].

**Figure 3:**
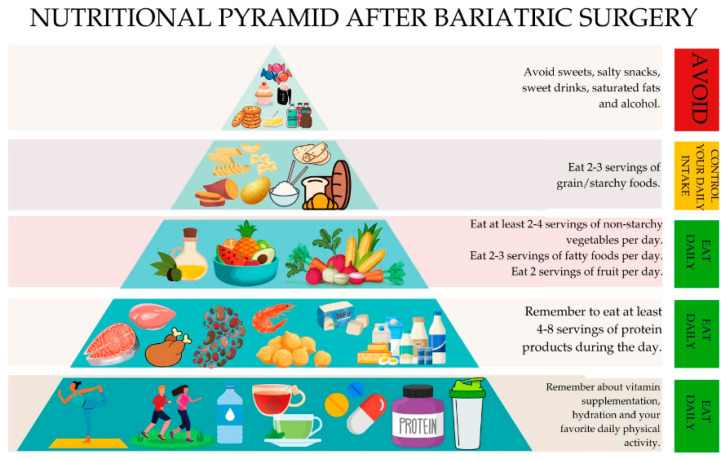
Nutritional pyramid after bariatric surgery based on [73].

In the later post-operative phase, a balanced diet containing all the necessary nutrients to maintain good health and a healthy body weight is recommended. At this point, it must be emphasized that there is little information in the literature on the diet recommended for long-term patients. The most commonly recommended long-term diets in the literature are the My Plate diet, the DASH diet, and the Mediterranean diet [3,95]. In addition, Moizé et al. developed the “bariatric food pyramid” in 2010—a nutritional guide for patients to better understand dietary recommendations and promote a healthy long-term postoperative eating pattern (Figure 3) [73].

Follow-up appointments with a qualified dietitian play an important role in helping the patient transition to a healthy lifestyle both in the short and long term after surgery. It is recommended that the patient attend follow-up appointments at 1, 3, 6, 9, and 12 months in the first year after surgery and at least once a year in the long term [96].

The long-term goals of surgical treatment of obesity during this period are:

- Permanent weight loss;
- Alleviation/reduction in the severity of diseases that are complications of obesity, e.g., high blood pressure, type II diabetes;
- Permanent change in diet (not a special diet) that enables systematic weight loss by:
- Reducing the risk of nutrient deficiencies;
- Reducing the risk of developing food intolerances;
- Preventing WR;

Preventing diet-related diseases.

### 5.3. Physical Activity After the Operation

**Figure 4:**
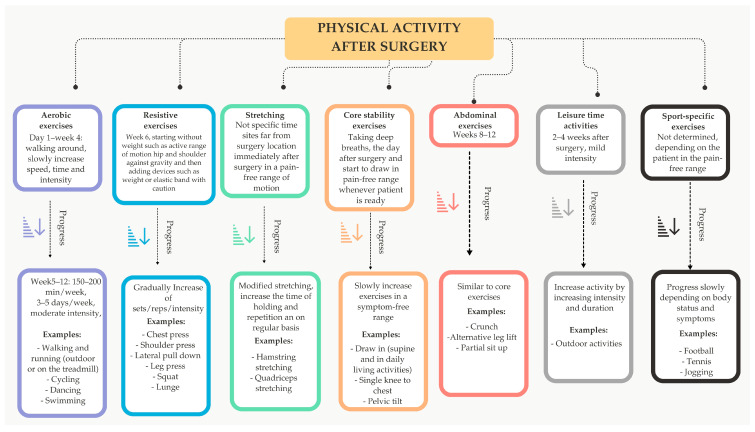
Physical activity after surgery based on [69].

In the first 4–6 weeks, patients can gradually increase their activity with the surgeon’s permission and under supervision. Do not lift weights for 6 weeks after surgery and do not perform any abdominal exercises for the first 8–12 weeks. In the first few months after surgery, patients should gradually increase their activity level under supervision but avoid high-intensity exercise (Figure 4). Therefore, bariatric patients should be well-guided to set realistic goals and consider their healing process to avoid damage or injury. At the same time, they should be encouraged to avoid long periods of rest and immobilization [69]. The long-term goal of training is to develop a habit of daily physical activity that will accompany the patient throughout his or her life [97]. Regular physical activity has a number of long-term health benefits, including maintaining a healthy body weight [69]. A 2019 meta-analysis confirms that BS significantly improves PA in patients with obesity up to 3 years after surgery [98]. After surgery, PA improves body composition by maintaining fat-free mass (FFM) and reducing FM, which could prevent WR in the long term [99].

### 5.4. Psychological Aspects of the Intervention

A patient is considered ready for BS when he or she has received approval not only from the surgeon but also from the dietician and the psychologist. The psychological assessment of the patient’s readiness for BS allows the diagnosis of possible comorbidities, especially psychological, that could interfere with the success of the treatment [75,76]. The ASMBS (American Society for Metabolic and Bariatric Surgery) recommends that the assessment of psychosocial behavior should be performed by a person with appropriate qualifications—a psychologist; social worker; psychiatrist; or nurse [3]. The aim is to provide the patient with a stable mental state, paying particular attention to psychological support both before and after the BS to increase long-term postoperative success and reduce the risk of complications [76].

**Figure 5:**
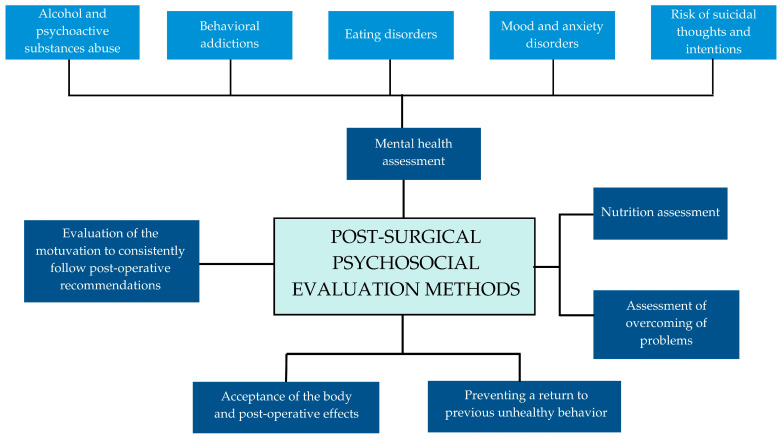
Post-surgical psychosocial evaluation methods.

The clinical interview is the basic diagnostic tool in this situation. The assessment of the mental state of patients after BS involves many aspects, which are illustrated in Figure 5.

The specialist should assess whether the patient is able to effectively change their eating behavior before and after the BS. It is recommended that the patient with obesity has access to postoperative psychological support. Studies show that this is associated with better outcomes after surgery [69].

### 5.5. Weight Loss and Weight Shifting Before Bariatric Surgery

Preoperative weight loss prior to BS is still controversial. Previous global and European guidelines [76] have recommended pre-op WL, while the most recent update of the ASMBS and IFSO guidelines [4] for the surgical treatment of obesity states that there is no scientific evidence to support the need for pre-op WL. Furthermore, the current IFSO consensus mentions that the use of pre-op WL remains controversial as the current published literature is inconclusive and pre-op WL should not be a mandatory requirement for patients who are suitable for BS [4,18,100]. More importantly, this practice is considered discriminatory, as it unnecessarily delays often life-saving treatment and promotes the progression of life-threatening complications of obesity and patient discontinuation of treatment. Bettini et al. conclude in their review that the evidence from randomized and retrospective studies does not currently support the hypothesis that pre-op WL could improve post-op WL after BS surgery [71]. According to the Decree of the Minister of Health of 12 August 2021 on the pilot program in the field of comprehensive specialist care for patients with severe obesity (KOS-BAR), which was implemented by a total of 19 centers in Poland, a multidisciplinary team should prepare an individual treatment plan and prepare the patient for BS as part of the 3–6 months of preoperative care. This includes a reduction diet that enables a reduction in body weight of at least 8–10% [101]. Pre-op WL can be achieved in a variety of ways, including dietary changes, increased physical activity, and pharmacotherapy. Unfortunately, it is common for patients to follow a restrictive, unbalanced, single-ingredient diet or even starvation diet during pre-op WL, which often leads to ND and malnutrition. These in turn can hinder the process of postoperative convalescence, impair wound healing, and also deepen in the postoperative period. In addition, healthcare providers should exercise caution when applying nutritional interventions in the acute pre-op WL, as some individuals are at high risk of malnutrition and/or sarcopenic obesity [102,103,104]. According to the recommendations of the Polish Society for the Treatment of Obesity (PTLO) and other foreign guidelines, measurement of BMI alone is not sufficient to assess the impact of obesity on health [67,68]. A helpful indicator is the measurement of waist circumference. In the adult European population, according to the IDF (International Diabetes Federation) diagnostic criteria, abdominal obesity is diagnosed at a waist circumference of ≥94 cm in men and ≥80 cm in women [105]. Recently, the European Association for the Study of Obesity (EASO), ASMBS, Obesity Canada et al. have published new global consensus statements on obesity, concluding that obesity treatment is about health, not weight, and WL should only be an outcome of obesity treatment [106].

**Figure 6:**
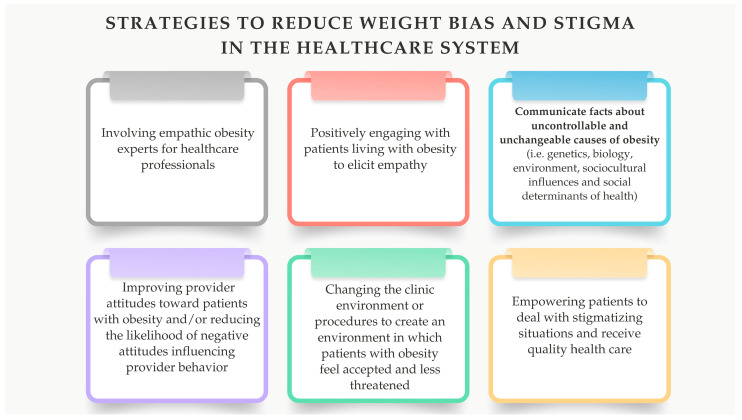
Strategies to reduce weight bias and stigma in the healthcare system.

The phenomenon of weight bias should also be mentioned in the context of WL. Weight bias can be experienced and maintained in two ways: (1) externalized weight bias (EWB), defined as negative attitudes directed externally toward others who are overweight and obese, and (2) internalized weight bias (IWB), defined as the internalization of publicly expressed stigmatizing weight biases that are important to oneself. People with obesity, including MO, frequently and commonly report stigma and discrimination based on their weight, and these experiences are associated with negative medical and psychological outcomes. Lawson et. al. indicate in their study that both constructs of weight bias (IWB and EWB) are associated with more severe depressive symptoms, lower self-efficacy in relation to one’s weight, and more eating disorder psychopathology, including loss of control over eating (LOC) and overvaluation of shape and weight [107]. Another study by Lawson et al., examined IWB and clinical correlates in adult patients with LOC eating after SG. The results suggest that IWB after SG is associated with greater eating disorder psychopathology, overvaluation of shape and weight, depression, and poorer self-reported mental health in patients with LOC eating [108]. These findings extend the literature by highlighting the clinical importance of IWB and eating disorder psychopathology in post-BS and the need for further research to better understand and more effectively assess and reduce internalized weight bias (Figure 6).

## 6. Challenges for the Long-Term Success of Bariatric Surgery

### 6.1. Compliance with Aftercare

Patients are usually advised to attend regular follow-up appointments after BS to monitor their progress and address any complications or problems [109,110]. The first year is crucial for postoperative FU care. It is usually recommended that patients are seen more frequently during this time. FU appointments usually take place at 2 weeks, 1 month, 3 months, 6 months, and 1 year, and then every year [111]. However, some patients have difficulty keeping these appointments due to various factors, including logistical difficulties, personal commitments, or a lack of motivation. The issue of patients not returning for FU after BS is of concern, as this can lead to poor quality of care and a lack of sufficient evidence of long-term BS outcomes [112,113]. Switzer et al. reported that 42 of 99 reviews (42.4%) did not meet McMaster criteria and 17 of 99 (17.2%) did not report FU outcomes [114]. It is estimated that the 5-year FU rate ranges from 29.6% to 54% [115,116].

**Figure 7:**
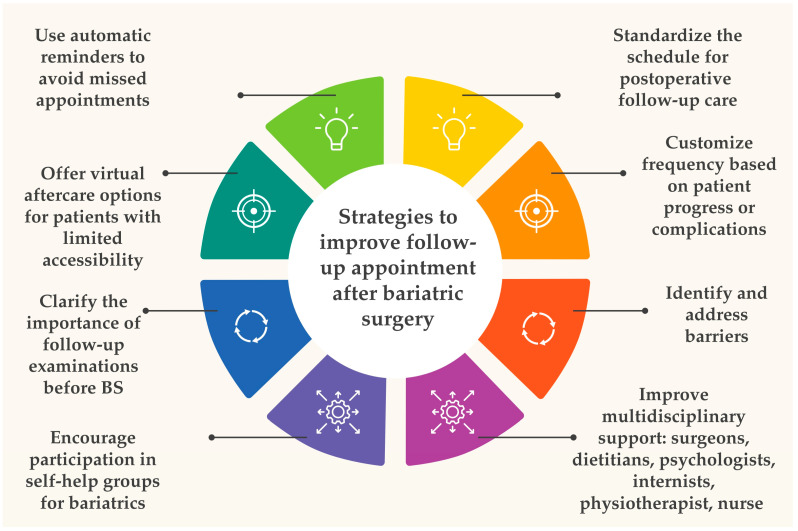
Strategies to improve follow-up appointments after BS based on [91,113,117,118,119].

Despite the scientific consensus [80] on the usefulness of long-term FU after BS, there is still a need to understand what causes patients to miss long-term FU appointments. In our opinion, the main problem is the insufficient education of patients about the importance of regular FU appointments and their role as important participants in the treatment process. This education should start before surgery and focus on raising patients’ awareness of the need for long-term FU appointments and the possible consequences of non-compliance. The second important aspect is the availability and quality of medical services. Many clinical centers are not logistically prepared for the long-term care of the growing number of post-BS patients, which leads to difficulties in organizing FU appointments. For the patient to recognize the value of regular check-ups, not only an individualized approach is needed but also improved access to specialized services such as counseling by a nutritionist, psychologist, or internist. Given the growing body of research and recommendations that emphasize the role of an interdisciplinary approach in the treatment of obesity, it is essential to establish centers of care with multidisciplinary teams as the gold standard. Such teams should include surgeons, dietitians, psychologists, internists, and other specialists who can work together to provide comprehensive patient care. In Figure 7, we present the key strategies that we believe can effectively improve patient engagement in long-term FU. These strategies include both educational efforts and a systemic approach to improving the accessibility and quality of healthcare services.

Below, we also outline key health challenges that may impact long-term patient compliance.

### 6.2. Nutritional Deficiencies and Nutritional Supplementation

The nutritional status of patients seeking BS is important. The risk of developing ND is influenced by preoperative, surgical, and postoperative factors. Poor preoperative nutritional status is a risk factor for both postoperative ND and metabolic complications. This emphasizes the need to identify and correct ND preoperatively as part of a comprehensive preoperative intervention [100]. Nutrition assessment by a registered dietitian with expertise in BS can help to obtain a comprehensive weight history, identify unfavorable dietary habits or patterns, and correct any micronutrient deficiencies before surgery, and after BS they can help to treat food intolerances, malabsorption, and micronutrient deficiencies [4,120].

The basis for a nutritional assessment or reassessment is a detailed nutritional history. This should also include an assessment:

- Holistic assessment of dietary habits and food/liquid intake;
- Targeted assessment of intake of identified sensitive food groups/micronutrient sources specific to the BS being performed;
- Assessment of adherence to routine micronutrient supplementation;
- Presence of gastrointestinal symptoms that interfere with food intake, such as reflux, vomiting, or other difficulties with eating;
- Behaviors toward food, such as aversion to food, fear of food, or signs of disturbed body image or eating habits [120].

**Figure 8:**
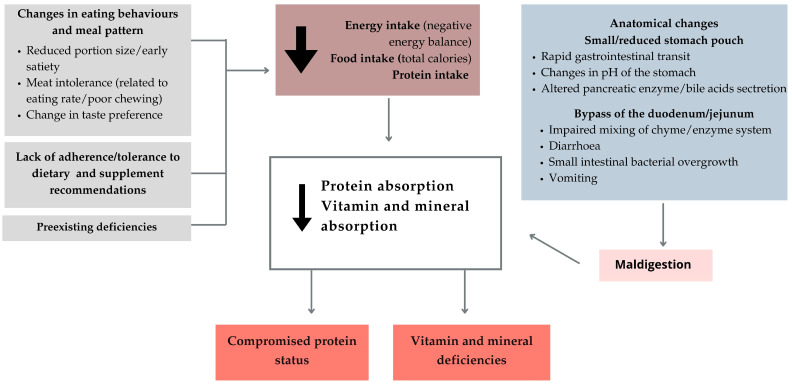
Anatomical, eating patterns, and behavioral changes that may contribute to the development of nutritional abnormalities after BS, based on [100].

Among the complications, ND deserves special attention. These can be caused by non-compliance with diet and supplement recommendations (Table 3), food intolerances after surgery, changes in the gut microbiome, changes in taste and eating behavior, or alcohol abuse after surgery (Figure 8). ND can have a wide range of clinical manifestations. As they can seriously affect the patient’s daily life and in some cases lead to life-threatening complications, biochemical tests that take into account vitamin and mineral levels in the serum are strongly recommended. They should be performed every three months during the first year after surgery and once a year thereafter [121,122]. In the subsequent phases of remote FU, patients should be continuously educated about proper nutrition. In surgical procedures where there is a risk of nutrient deficiency, long-term supplementation with vitamins and micro- and macronutrients is indicated [86,123].

The common perception is that a patient with obesity is a well-nourished person. This is a misconception because excessive body weight can mask an ND. The diet of a patient with obesity is very often rich in saturated fatty acids and trans-isomers of polyunsaturated fatty acids and simple carbohydrates, while being deficient in complex carbohydrates, fiber, minerals, and vitamins [121]. The most common NDs in bariatric patients include protein, B vitamins (especially B_1_ and B_12_) [125], some fat-soluble vitamins, as well as zinc, selenium, iron, and copper. In addition, every bariatric patient should be prescribed a long-term mineral and multivitamin supplementation depending on the procedure. In patients with documented micronutrient deficiencies, supplementation should be individualized [122]. Special attention should be paid to BS patients who become pregnant to avoid macronutrients and micronutrient deficiencies. Therefore, careful assessment and monitoring are of great importance, including laboratory screening in each trimester, depending on the surgical procedure (i.e., iron, folic acid, vitamin B12, vitamin D, calcium, fat-soluble vitamins, zinc, and copper) [126]. 

A systematic review examining the association between the type of BS (Laparoscopic Adjustable Gastric Banding, RYGB, SG) and diet quality at least 1 year after surgery concluded that reduced energy intake after surgery, as well as inadequate micronutrient and protein intake and excessive fat intake, may contribute to ND and cause WR. It has also been noted that patients do not adhere to vitamin and mineral intake recommendations and that medical staff do not adequately monitor the intake of micro- and macronutrients in patients’ diets [85]. Some studies indicate decreased serum concentrations of B vitamins, which could be related not only to their poorer absorption from the gastrointestinal tract but also to low dietary intake, confirming the key role of the patient’s cooperation with the dietitian [127,128]. To minimize the risk of deficiency, the patient should be encouraged to choose high-quality foods that provide proteins of high biological value, i.e., containing all essential amino acids. To prevent excessive loss of lean body mass, regular monitoring of the patient’s nutritional status and diet by an experienced dietitian is required. Protein supplementation is indicated in the postoperative phase. It should also be borne in mind that some patients with obesity in the preoperative phase are likely to have a protein deficiency, usually due to an unbalanced diet. Therefore, total protein levels, serum albumin, and prealbumin should be determined for each patient during the qualification process [129]. It has been shown that preoperative assessment and correction of deficiencies can lead to better outcomes in people after BS. In addition, adherence to postoperative dietary recommendations and participation in regular FU visits significantly reduce the risk of ND. Long-term or even lifelong monitoring of health and nutritional status and appropriately tailored interventions are crucial [126].

### 6.3. Weight Recurrence After Bariatric Surgery

A review of the literature shows that 20–30% of bariatric patients do not reach their target weight in the first few years after surgery. However, 25–35% of patients regain weight within 2–5 years after surgery [130]. Unfortunately, these results are even less optimistic in the long term. Further, WR in operated patients leads to frustration, depression, and recurrence of obesity-related diseases [131]. WR after BS is associated with a decrease in quality of life and satisfaction with the procedure. Determining when WR is abnormal is critical for setting appropriate patient and clinician expectations and for planning interventions to maximize WL [132]. The etiology of excessive WR is usually complex and related to anatomic factors, long-term postoperative complications, and somatic disorders [133]. However, postoperative WR is largely due to a return to old eating habits. The mechanism of WR after BS may be exacerbated by the patient’s unrealistic expectations and wishful thinking that surgery will prevent WR. The lack of a thorough diagnosis and education about the role of eating and the lack of early introduction of adaptive behaviors that regulate the affective state increases the risk of returning to old habits and regaining body weight [94]. There is no standardized definition for WR after BS, resulting in varying recovery rates of regain, ranging from 6% at two years to 76% at six years postoperatively [133]. In a study examining rates of WR 5 years after BS, it was noted that it is difficult to find a standardized categorical definition for clinically significant WR. This emphasizes the need for additional research on the clinical significance of WR [134]. Despite the lack of a standardized definition, it is evident that post-BS WR is a multifactorial condition affecting many patients, highlighting the need for individualized and comprehensive assessment by a multidisciplinary team [135,136]. The most common definition found in the literature is a weight gain of more than 10 kg from nadir (the lowest post-BS weight) [137].

Several risk factors for WR after BS can be identified as patient-related factors: higher baseline BMI, infrequent attendance at specialist visits before surgery, socioeconomic status, lifestyle, noncompliance with diet and physical inactivity, mental illness during treatment, male gender, metabolic imbalance, higher ghrelin levels after BS, genetic and anatomical factors [42,128,138]. Surgical factors for WR are the diameter of the gastrojejunal stoma and the gastric volume after SG, the dilatation of the gastric pouch, and the choice of surgical technique (restrictive or malabsorptive) [42,139,140]. While WR can be attributed to anatomical and surgical causes in a small percentage of cases, the main causes of WR appear to be postoperative increased food intake due to increased appetite and maladaptive or disordered eating behavior, inadequate physical activity, and psychosocial stress [134]. Another aspect that influences WR is the extent of the decrease in FFM, especially skeletal muscle mass. The less muscle mass is lost in the initial phase of WL, the greater the likelihood that the lower body mass will be maintained in the long term [141,142]. The FFM mainly determines the basal metabolic rate (BMR), the main component of daily energy expenditure, and the concentration of leptin, a key hormone for body weight homeostasis, whose action regulates energy expenditure and food intake [141]. In addition, FFM appears to be negatively correlated with energy intake and portion size [143]. Another aspect is that there is some evidence that weight loss can lead to a disproportionate reduction in resting metabolic rate (RMR). The reduction in RMR after weight loss could be related to the high FFM lost during the WL period. After BS, many patients do not pay attention to their body composition. They are usually preoccupied with WL and not whether they are losing FM or FFM. An abnormally low RMR may predispose surgical patients to WR. In their study, Faria et al. found that patients with WR had a lower RMR than healthy-weight patients. They suggested that to promote weight loss and prevent weight gain, all healthcare professionals should promote mechanisms that increase the patient’s total energy expenditure by increasing the consumption of protein- and fiber-rich foods and reducing the consumption of high-fat foods, increasing the frequency, duration, and intensity of physical activity, decreasing body fat percentage, and increasing FFM [144].

Fortunately, there are several scientifically supported strategies to address this problem. Patients who regain weight should undergo a comprehensive evaluation by a multidisciplinary team to determine the causes and initiate appropriate individualized treatment, which may include treatment of postoperative complications, nutritional and behavioral counseling, and pharmacologic and surgical interventions [92,145,146,147]. Lifestyle interventions such as diet, exercise, and behavior modification are fundamental to the treatment of WR, although their effectiveness is limited [134]. Postoperative cognitive-behavioral interventions are important to prevent WR and promote adherence to postoperative recommendations and lifestyle modifications [92]. In addition, pharmacotherapy may enhance the effectiveness of lifestyle interventions in the treatment of WR after BS [92]. Initiating pharmacotherapy at the time of weight plateau may maximize WL outcomes after BS [92,135]. In addition, more attention should be paid to long-term follow-up, which should be based on collaboration between the patient and the entire therapeutic team. Therapeutic education of patients, and encouraging them to participate in their treatment, plays a key role in monitoring lifestyle changes after BS [148]. In conclusion, further research is needed to gain a better understanding of the behavioral and biological correlates of weight gain and to provide a rational basis for the development of better prevention and treatment strategies.

### 6.4. Revisional Surgery

Redo BS, also known as bariatric revision surgery (RS), is a procedure performed in patients who have previously undergone BS but have experienced WR, insufficient weight loss (IWL), or complications [149,150,151]. This surgery is usually recommended when the original WL goals have not been achieved or when complications occur (e.g., GERD). The procedure may involve a revision of the original surgical procedure, a change from one surgical WL method to another, or an adaptation of the existing procedure. When it comes to the revision of SG, the most popular choice is switching to RYGB, as it effectively addresses GERD and improves post-op WL [152]. The most common indication for revision is GERD. It should not be overlooked that de novo GERD develops in 48% of patients during a follow-up period of 8.5 years after SG [153]. Matar et al. reported in a meta-analysis that 52% of conversions are due to WR/IWL [154]. However, it is important to note that RS is more complicated and associated with a higher complication rate [155,156,157,158]. RYGB is most commonly converted due to the presence of marginal ulceration (MU) and is significantly more common in cases that were already the conversion of a failed SG (S-RYGB) [159]. Reported rates of MU development vary from 0.6% to 25% [160,161,162]. OAGB is most frequently revised due to GERD. The rate of conversion is estimated to be 1.2–8% [163,164]. In summary, the most common reasons for conversion are WR/IWL and GERD. WR can be due to various reasons, such as a change in diet and exercise habits, hormonal fluctuations, psychological factors, or surgical complications [165,166].

### 6.5. Eating Disorders

**Figure 9:**
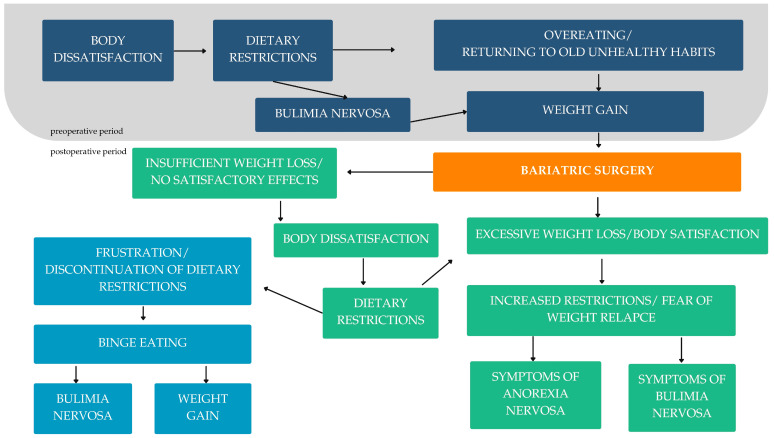
Summary of the EDs problem in the context of BS.

Disordered eating behavior has been reported to be very common in BS patients both before and after surgery. The most common include grazing (26%), emotional eating (38%), sweet eating (43%), loss of control (61%), binge eating (64%), and food cravings (90%). Many patients also suffer from complications associated with EDs, including gastrointestinal distress or ND [167]. Patients’ concomitant eating habits may play an important role in the effects of postoperative WL. In addition, the presence of a history of binge eating disorder (BED) did not appear to predict a difference in WL two years after surgery, but this area remains controversial and requires further investigation [64]. It is known that maladaptive eating behaviors, particularly loss of control and binge eating, are factors that may impede successful long-term WL. LOC (i.e., feeling unable to stop eating or unable to control how much one eats) is a central feature of binge eating (i.e., eating an objectively large amount of food that is accompanied by a feeling of loss of control) and BED, which was introduced in the fifth edition of the Diagnostic and Statistical Manual of Mental Disorders—DSM-V and is characterized by recurrent episodes of binge eating without regular compensatory behaviors [168]. Overeating may have medical causes (e.g., neurochemical or endocrine causes related to damage to the satiety center), a lack of knowledge and awareness of the principles of proper nutrition, a lack of stress management, or, finally, the co-occurrence of EDs. Especially in the first and last cases, the patient has limited ability to control his eating behavior and is often unaware of the true cause of his difficulties. Only if the primary cause of overeating is correctly diagnosed and treated does the patient have a chance of effectively and permanently reducing their body weight [169]. Emotional disturbances related to the feeling of LOC, even when subjectively eating large amounts of food, are common in candidates for surgical treatment. Eating smaller amounts over longer periods of time has also been described before and after surgery. This eating behavior is commonly referred to as “grazing”. Binge eaters who eat prior to surgery are at high risk of engaging in “grazing” after surgery [170]. In addition, “grazing” has been associated with poor weight outcomes in bariatric patients [171]. In a study by Saunders R. [172], more than 60% of BED patients reported recurrent grazing in the first 6–12 months after surgery, and 44% were considered uncontrolled eaters. More than one-third of the BED group met criteria for both postoperative eating behaviors at baseline [172]. Only when the primary cause of overeating is correctly diagnosed and treated do those affected have a chance of effectively and permanently reducing their body weight [169]. It has been observed that patients who attended fewer clinic appointments were more likely to report BED before surgery and had lost less weight [170]. The current literature focuses more on LOC and binge eating disorder, as both conditions are often associated with obesity. Other EDs such as anorexia nervosa (AN), bulimia nervosa (BN), and atypical EDs associated with BS have recently gained interest. However, due to differences in assessment and study design, there is still no consensus in the literature on the prevalence or incidence of these EDs in postoperative bariatric patients. Therefore, they may be overlooked or misdiagnosed by the bariatric team, especially if the WL corresponds to the expected WL after BS or if the BMI again remains in the overweight or normal weight category. Furthermore, the trade-off between the conflicting goals of WL for BS and the weight gain required to treat a restrictive ED raises additional issues beyond what is routinely expected when both conditions are treated alone [173]. In the systematic review and meta-analysis, the researchers investigated the relationship between BS and the development and recurrence of EDs. The main problem in analyzing the available literature was the lack of a gold standard for diagnostic methods. Therefore, it was difficult at this stage to determine the association between BS and the development of EDs. However, this possibility should not be excluded, as other surgical procedures have already been shown to be associated with the development of mental disorders, e.g., cardiac surgery, gastrointestinal cancer surgery, or liver transplantation [174]. The restrictive eating regimes recommended after BS are closely associated with extreme WL. Portion sizes are significantly reduced, and patients are often encouraged to weigh their food and strictly control their calorie intake. Certain foods (e.g., very sweet foods) and certain eating habits (e.g., eating too fast or not chewing enough) are discouraged. The changes after surgery (i.e., rapid weight loss and dietary restriction) may trigger or promote the development of a restrictive “AN-like” eating disorder characterized by greater than usual weight loss after surgery, fear of regaining weight, dietary restriction, and disturbed self-perception of shape and weight. As this area is poorly researched, the prevalence of restrictive eating disordered behavior after BS is unknown, although it is becoming increasingly common [175]. In Figure 9, we summarize the problem of EDs in the context of BS.

According to Katrina Parker et al. [176], there are 4 main difficulties in diagnosing EDs in the post-BS population:

1. The most commonly used scales have been developed for traditional eating disorders (i.e., AN and BN), which have not been psychometrically evaluated in the post-BS population or only to a very limited extent.
2. The altered gastrointestinal system following surgery creates a unique eating context that may not be validly assessed with existing measurement tools.
3. Physiologic restriction after surgery likely affects the ability to eat abnormally large amounts of food, as required for a diagnosis of binge eating, and may also alter perceptions of LOC, as some patients believe that surgery decreases control over eating. Postoperative recommendations, such as reducing portion sizes, eating slowly, and chewing thoroughly, are similar to some EDs assessed with existing measurement tools.
4. If postoperative dietary recommendations are not followed, maladaptive eating symptoms such as vomiting, regurgitation, diarrhea, and other gastrointestinal complications very similar to disordered eating behaviors (e.g., vomiting as purging) may develop. Unlike psychological-induced eating disorders, these symptoms are the result of a physiological and behavioral maladaptation to the altered gastrointestinal system.

Taken together, these challenges mean that existing measurement methods may not adequately capture the extent of the EDs and fail to recognize the differences in functioning between psychologically driven Eds and behaviorally driven Eds in the postoperative population [176].

Below are some warm-up exercises and clinical considerations for EDs in overweight individuals:

- Recent fluctuations in body weight
- Dietary restrictions for non-medical (e.g., allergy) or medical reasons
- Food consumption or restriction to regulate emotions
- Compulsive exercise
- Body image concerns
- Depression, anxiety, substance abuse
- Presence of ND [177].

In summary, EDs and dysfunctional eating behaviors should be a mandatory topic of preoperative assessment and FU visits as they impact long-term postoperative outcomes.

### 6.6. Alcohol Abuse and Other Addictions

Despite the undeniable benefits of surgical treatment of obesity, there are also negative effects associated with an increased risk of impulse control disorders, including alcohol dependence. Therefore, many recommendations suggest limiting or stopping alcohol consumption and maintaining this habit for a prolonged period after surgery [3,122]. According to ASMBS recommendations, any patient who is a candidate for BS and who is diagnosed or suspected of alcohol abuse should be screened for mental health by qualified medical personnel prior to surgery. Alcohol screening is required on both the preoperative and postoperative checklist for both candidates and patients after surgery [3]. Preoperative risk factors include genetics, male gender, age, history of alcohol consumption, smoking, and low social support [178]. In a cohort study, no significant association was found between postoperative alcohol use disorder (AUD) and preoperative mental health, binge eating, depressive symptoms, or treatment for psychiatric or emotional problems in the past year [179]. When it comes to possible predictors of alcohol abuse after BS, it is worth mentioning the concept of “addiction transfer” or “cross-addiction”, which appears frequently in both the lay media and some scientific journals. It assumes that people who develop an alcohol problem after BS previously had an “addiction” to food, which “transfers” to alcohol after surgery. However, this hypothesis has not been confirmed. Furthermore, long-term studies on alcohol problems after BS consistently show that these problems usually develop 1 to 2 years after surgery. If patients had a need to replace food and eating with some other type of substance or behavior, we would expect this need to be most pronounced in the first few months after surgery, when patients are most limited in their intake and tolerance for highly palatable foods. Indeed, most patients are able to eat significantly larger quantities and a greater variety of foods 1–2 years after BS, so the need to satisfy unsatisfied food cravings is likely to be less rather than more pronounced during this period [178]. Patients after RYGB and SG are at risk for AUD due to impaired alcohol metabolism in the postoperative phase [3]. Alcohol metabolism and alcohol dependence are recognized problems that are more common in patients who have undergone malabsorptive BS. For example, in women who have undergone RYGB, two alcoholic drinks are equivalent to consuming four alcoholic drinks [180]. In a cohort study, 1945 patients who had undergone BS had a significantly higher risk of alcohol use disorder 2 years after surgery than in the year before surgery. This was attributed to male gender and RYGB [179]. In the study, patients who had undergone BS had a significantly higher risk of developing alcohol abuse than patients who had not undergone a restrictive surgery. It has already been shown that patients who have undergone gastric bypass reach higher peak alcohol levels and take longer to reach zero after alcohol consumption than non-operated controls with obesity and patients before surgery [181]. Furthermore, in the Swedish SOS study, the proportion of people with at least moderate alcohol consumption was highest among those who had undergone gastric bypass surgery. This study shows that alcohol consumption decreases in the first year after surgery, which underlines the importance of long-term FU [182]. It is known that problems with alcohol tend to occur later after surgery [162]. However, a report by Acevedo et al. showed that SG is similar to RYGB in terms of negative effects on patients’ alcohol consumption. Indeed, in these patients, faster and higher peak blood alcohol concentrations lead to underestimation of alcohol concentration by breath alcohol analyzers [183]. Patients considering BS should be informed of postoperative AUD risk factors, including the type of surgery. In addition, preoperative and postoperative care should include alcohol and AUD screening, assessment, and possible referral to treatment [184]. Furthermore, postoperative AUD is a potential side effect, and in addition to the general negative effects of alcohol on health, BS patients suffering from AUD are at greater risk of regaining weight or suffering from ND [182]. Ultimately, AUD can lead to significant long-term health consequences in patients undergoing BS, including an increased risk of serious illness, higher rates of hospitalization, and poorer outcomes. The association between BS and AUD is thought to have a multifactorial basis, i.e., physiological and psychological [178]. Although there are no clear explanations for the mechanism of AUD, it is important to emphasize the risk factors and alcohol consumption behaviors prior to BS and to educate candidates about the risks associated with surgical treatment of obesity, especially in restrictive and exclusionary procedures.

## 7. Conclusions

In addition to adhering to lifestyle modification recommendations and guidelines for patients’ lifestyle changes after BS, it is important to recognize and address the potential difficulties that patients may face during their treatment, especially with regard to long-term follow-up and recurrence of obesity after surgery. Therefore, strategies need to be constantly updated and individually assessed to meet the unique needs of each patient. More emphasis should be placed on educating both patients and healthcare professionals about the difficulties and complications following surgery. This can help to create medical centers where specialists pay special attention to multidisciplinary care after surgery by educating patients and creating the possibility of continuous contact with specialists according to their individual needs. In addition, further research is needed to better understand why surgical treatment does not always produce the expected results. This underlines the need for a differentiated and patient-centered approach to the treatment of obesity.

## Figures and Tables

**Table 1:** Stages of diet after BS based on [3,80,81,82].

Stage of Diet After BS	Stage 1 Clear Diet	Stage 2 Full Liquid Diet	Stage 3 Purred Diet	Stage 4 Soft Solid Diet	Stage 5 Normal Diet
Postoperative days *	1–2	3–7	8–14	15–21	Starts 4–5 weeks after BS
Characteristics	Only clear liquids with body temperature (non-carbonated, no sugar, co-calories, no caffeine, and no fiber). Min 1.5 L fluids per day to prevent dehydration. For the first 2 h after surgery, 15 mL of liquid should be eaten every 30 min for the first 2 h, and increase to 15 mL every 15 min for the rest of the day.	Foods with semi-liquid consistency: cocktails, soups, creams, yogurt, milk soups. It should be remembered that fluid portions should depend on the individual tolerance of each patient. However, a single serving should not exceed ½ cup. Patients should be encouraged to consume 120–170 mL of liquids every hour.	High-protein foods, grated, blended, and soft food. Encourage patients not to drink with meals and to wait ~30 min after each meal before consuming fluids again.	Solid food with a soft consistency, e.g., crushed with a fork, chopped, or grated. Delicate thermal treatment, e.g., cooking, steaming, choking	Beginning of introducing meals without changing the consistency of products. Used long-term.

* The speed of diet expansion can be extended by a few days depending on the patient’s tolerance.

**Table 2:** Nutritional recommendations after BS.

Topics	Recommendations
Eating habits	As soon as patients can tolerate 0.5 cups of food in one sitting, daily intake should be limited to 5–6 meals (according to the postoperative stage). Patients should get at least 20 min for every meal and chew every food slowly and adequately to prevent blockage. Patients should be encouraged to include protein in every meal. Patients should chew small bites of food thoroughly before swallowing.
Protein intake	min. 60/80 g/d or 1.5 g/kg of ideal body weight/day
Carbohydrates intake	min. 50–130 g/d
Fiber intake	14 g/1000 calories
Fat intake	20–35% of the daily caloric intake
Fluid intake	>1.5 L/d; avoid drinking for 30 min. before/after eating solid food

**Table 3:** Vitamin and mineral prevalence of deficiency after BS and recommended supplementation based on [3,86,124].

Vitamin/Mineral	Prevalence of Deficiency After BS	Recommended Supplementation to Prevent Deficiency	
		SG	RYGB
Vitamin A	Up to 70% of patients within 4 yr. post-surgery	5000 IU/d	5000–10,000 IU/d
Vitamin D	Up to 100% of patients	3000 IU, until blood levels of 25 (OH) D are greater than sufficient (30 ng/mL)	
Vitamin E	Uncommon	15 mg	
Vitamin K	Uncommon	90–120 µg	
Vitamin B_1_	1–49% depending on procedure and post-surgery time frame	12 mg/d For at-risk patients: 50–100 mg	
Vitamin B_12_	At 2–5 yr. post-surgery time frame After RYGB: <20% After SG: 4–20%	350–500 µg	
Foliate (folic acid)	Up to 65% of patients	400–800 mg/d 800–1000 mg/d for women of childbearing age	
Iron	3 mo.–10 yr. post-surgery time frame After SG: <18% After RYGB: 20–55%	Males and patients without a history of anemia: 18 mg Menstruating women: 45–60 mg	
Calcium	Up to 100% of patients	1200–1500 mg	
Zinc	Up to 19% of patients after SG Up to 40% of patients after RYGB	8–11 mg	8–22 mg
Copper	10–20% in patients post-RYGB	1 mg	1–2 mg
Magnesium	32%	400 mg	
Vitamin B_2_	nd	3.4 mg	
Vitamin B_3_		40 mg	
Vitamin B_5_		20 mg	
Vitamin B_6_		4 mg	
Biotin		60 µg	
Vitamin C		120 mg	
Selenium		140 µg	
Manganese		4 mg	
Chromium		120 µg	
Molybdenum		50 µg	

## Data Availability

The original contributions presented in this study are included in the article. Further inquiries can be directed to the corresponding authors.
